# Emerging Insights into Antibiotic-Associated Diarrhea and *Clostridium difficile* Infection through the Lens of Microbial Ecology

**DOI:** 10.1155/2008/125081

**Published:** 2008-12-04

**Authors:** Seth T. Walk, Vincent B. Young

**Affiliations:** ^1^Division of Infectious Diseases, Department of Internal Medicine, University of Michigan Health System, Ann Arbor, MI 48109, USA; ^2^Department of Microbiology and Immunology, University of Michigan, Ann Arbor, MI 48109, USA

## Abstract

Antibiotics are the main, and often only, clinical intervention for prophylactic and active treatment of bacterial infections in humans. Perhaps it is not surprising that these drugs also shift the composition of commensal bacteria inside our bodies, especially those within the gut microbial community (microbiota). How these dynamics ultimately affect the function of the gut microbiota, however, is not fully appreciated. Likewise, how antibiotic induced changes facilitate the outgrowth and pathogenicity of certain bacterial strains remains largely enigmatic. Here, we discuss the merits of a microbial ecology approach toward understanding a common side effect of antibiotic use, antibiotic-associated diarrhea (AAD), and the opportunistic bacterial infections that sometimes underlie it. As an example, we discuss how this approach is being used to address complex disease dynamics during *Clostridium difficile* infection.

## 1. INTRODUCTION

The human colon contains the most abundant and diverse assemblage of bacteria in the body. Symbiotic interactions with and within this complex community are now recognized as important predictors of human health. Aberrant community structures are associated with complex diseases like obesity, irritable bowel syndrome, and immune dysfunction. Antibiotic administration can disrupt the colonic ecosystem, which, in turn, leaves patients vulnerable to gastrointestinal disease. Diarrhea is a common manifestation of antibiotic-mediated disturbance and can result from altered function of the disrupted microbiota, direct effects on host tissue, and colonization by opportunistic organisms that invade the altered microbial community. Here, we review the relevant microbial ecology of antibiotic-associated diarrhea with an emphasis on bacterial community dynamics during *C. difficile* infection.

## 2. COMMONALITIES AND ASSUMPTIONS FOR GI TRACT MICROBIAL ECOLOGY

When initiating a discussion of the microbial ecology of the gastrointestinal (GI) tract, it is important to review some of the common areas and assumptions investigators used when studying this ecosystem. First, the proportion of uncultivable bacteria in the GI tract is high (∼60%–80%). Initially, culture-based surveys of the gut microbial successfully isolated and characterized large numbers of the bacterial morphotypes (i.e., distinct cellular forms) present in human feces [1, 2]. However, recent surveys based on DNA sequencing have indicated that the vast majority of genetically distinct organisms have not been isolated by culture techniques [3]. These relatively new sequence-based approaches in combination with robust bioinformatics provide the framework to explore a vast amount of genetic diversity. It is now feasible to survey nearly all of the genetic information in a given system, and this ability has ushered in a new area of research, referred to as metagenomics [4]. The field is still in its infancy, and much of the data continue to be open for interpretation. It is important to note that the currency for GI tract microbial ecology in the metagenomic era is the abundance and distribution of targeted DNA sequences and not actual organisms or randomly sampled genomes of organisms. The amplification, cloning, and sequencing of certain loci, such as the highly conserved 16S rRNA locus, are the tools used to study the phylogenetic signal contained in the metagenome, and this is different than classical metagenomics, where one seeks to analyze the functional and sequence-based diversity contained in all microbial genomes of communities [4, 5]. Lastly, we draw attention to an early few studies that use culture-based approaches, but will put these data into a metagenomic context.

There are measurable, statistical, and real differences (i.e., not all the detectable differences are biologically significant) between the bacterial communities throughout the human body (skin, mouth, vagina, GI tract, etc.). Studies have shown regional differences in microbial composition throughout the mammalian GI tract in both the longitudinal (i.e., stomach to small intestine to large intestine) and axial (i.e., mucosal associated to mucus to lumen) directions [6–8]. For further discussion on this topic, see the recent review by Peterson et al. [7]. Currently, most studies circumvent the practical and ethical problems associated with direct intestinal sampling (e.g., via colonoscopy and biopsy) by using feces as a proxy [9]. Many of the studies reviewed here do the same and regard the bacterial community in feces as representative of the gut microbiota as a whole, with the caveat that existing spatial community differences may result in a biased representation. For example, total anaerobe counts were found to be 100 times lower in the human cecum compared to feces [10].

Lastly, it is generally assumed that the abundance and distribution of an organism (16S rRNA gene sequence) and broader taxonomic groups of organisms (sequences grouped based on percent similarity and called operational taxonomic units or OTUs) are important. The abundance and distribution of OTUs are often called community structure. As we will discuss in detail below, there are observable patterns in the gut microbiota under certain conditions. Some taxonomic groups are very abundant, while others are at such low abundance that they can only be detected using highly sensitive and specific molecular techniques. Most studies look for community structure and try to assess the underlying mechanisms that caused it (disease, diet, drug effect, etc.). While this may at first seem logical and perhaps trivial, it is currently not well understood what these patterns really mean. For example, what OTUs should be used to assess structure? At the phylum level, patterns may be clear, but at the species level, where functional variation is driven by evolutionary processes, the structure may not be statistically different from a random assemblage (due, in part, to the lack of a universal bacterial species concept [11]). Currently, a challenge for microbial ecologists is to understand dynamics with respect to the functional attributes of bacterial communities and not only through the lens of taxonomy.

## 3. NORMAL GUT MICROBIOTA

The human colon is typically associated with 10^11^ to 10^12^ bacterial cells per gram of contents, and new estimates using genetic diversity suggest that the gut ecosystem holds 15000–36000 different species [9, 12, 13]. Colonization normally begins at birth, and a variety of bacteria can be detected in infant stools within the first few days after vaginal delivery [14]. Among the first gut bacteria to colonize infants were *Escherichia coli* and *Staphylococcus aureus* [15, 16]. These studies used culture-based methods to show that abundance was highest at about one week after birth and decreased 1–3 orders of magnitude within the first year, suggesting that the abundance of early bacterial colonizers is subsequently shifted by the growing biologic complexity of this system. Recently, nonculture-based data supported these findings and showed that multiple shifts occur among different taxonomic groups over the first 200 days of life [17]. Also, the gamma-Proteobacteria, to which *E. coli* belongs, appear to be the dominant members in these infant's GI tract. It is interesting to note that *E. coli* was initially discovered in 1884 and studied by the famous German pediatrician Theodor Escherich because of its presence in “normal” infant microbiota and because of its beneficial effects on digestion [18].

Defining normal gut microbiota is challenging because of the compositional heterogeneity that exists between hosts [19]. Most phylotypes (suspected species) are unique to the individual being sampled [3]. At broader taxonomic levels, a consistent community structure is often observed, leading to the conclusion that the gut is dominated by members of a few bacterial phyla (Firmicutes, Bacteroidetes, Actinobacteria, and Proteobacteria). The human gut is described as “exclusive” because there are more divisions (phyla) of bacteria and archaea known to exist on earth than what is typically sampled from human subjects (currently the Silva 16S rRNA database has 115 bacterial divisions of which only 10 have been sampled from humans) [7].

The bacteria in our GI tract are important for certain aspects of human health, and there are clear mutualisms between human and bacterial cells [20]. Not surprisingly, our immune system defends against negative symbiotic interactions based on prior exposure and also on stimulating mechanisms like breast feeding and vaccinations (prior exposure to living cells is not always necessary for an effective immune response). Some of the traits that make us human also dictate the structure of the gut community, as the microbiota of conspecific relatives (same species of humans, primates, and nonprimates) was most similar to each other in a recent study [21]. There are few data that describe the community structure of the GI tract microbiota in healthy individuals and this limits our ability to formulate generalities on the normal state. However, if we are to consider the healthy human gut as a theoretically-based community, where a consistent structure is defined and used to test hypotheses, then the microbiota of individuals should converge upon a similar structure under similar conditions [22]. In the absence of convergence, we are left to the study of stochastic events and patterns that are best explained by random walk models, where species traits do not correlate with the abundances along environmental gradients (for more on the theoretical issues concerning community analysis, see Tilman [22]).

Because of the low degree of similarity between individuals, changes in the gut microbiota are typically measured by shifts in structure. For example, a cohort study of 1032 infants showed that breast-fed infants have a consistently different bacterial composition than bottle-fed infants [23]. Based on real-time PCR and OTU specific probes, formula-fed infants (*n* = 232) were colonized by *E. coli*, *C. difficile*, *B. fragilis*, and lactobacilli more often than breast-fed infants (*n* = 700). Similar comparative studies have shown associations between an altered gut microbiota and a number of human diseases, including obesity [24], Crohn's disease [25], irritable bowel syndrome [26], and allergies [27]. It is clear that our understanding of the normal gut microbiota is limited and just beginning, but comparative studies like these illustrate a novel ability to describe the microbial ecology that underlies many complex diseases.

## 4. ANTIBIOTICS INCREASE HOST SUSCEPTIBILITY TO PATHOGENS

One measure of ecosystem stability, in terms of maintaining function [28], is the ability to resist invasion and subsequent dominance by immigrating organisms. For the gut ecosystem, antibiotic therapy represents a strong perturbation that shifts the relative proportion of community members, allowing opportunists to establish [29–32]. Antibiotic therapies exclude members of the community by eradicating them directly or indirectly by breaking necessary mutualistic interactions [33]. During such events in murine models, the community structure was disrupted and enteric pathogens reached high numbers [34, 35]. Similar observations underlie the proposed colonization resistance or barrier function, provided to the host by the gut microbiota [32, 36, 37], preventing the ingress of pathogens into the gut ecosystem.

Many details about the colonization resistance function of the microbiota have yet to be tested, but it is clear that shifts in the gut microbial community structure are permissive to the establishment of certain pathogens. For example, *Vibrio cholerae* does not normally cause disease in conventional guinea pigs, but it established and caused severe disease after disruption of the microbiota by pretreatment with streptomycin [38]. Similarly, it has been shown that mice with a conventional gut microbiota require a much higher infective dose (10^9^ colony forming units per mL, CFU/mL) for colonization by a gram-negative bacterium compared to antibiotic treated mice (10^2^ CFU/mL) [39]. The mechanisms behind colonization resistance in humans are topics of ongoing research, but the gut microbiota in animal models has been shown to (i) utilize essential nutrients before they are available to invading bacteria (resource limitation), (ii) limit access to attachment sites (space limitation), and (iii) produce inhibitory substances [40].

Many factors, including drug dose, route of administration, absorption, and host inactivation, dictate the intensity of antibiotic effects on the gut microbiota (see review by Sullivan et al. [32] for specific effects of commonly used drugs). A number of culture-based and nonculture-based molecular techniques have been used to follow bacterial community dynamics in humans upon exposure to antibiotics. Often, specific groups of OTUs are singled out with specific probes. Temporal effects of antibiotic treatment were recently shown among members of the Bacteroidetes division using culture techniques and genetic fingerprinting (rep-PCR) [30]. During a case-control study of subjects taking capsules of 150 mg clindamycin (orally), each individual was sampled prior to antibiotic treatment and at set time points throughout the following 2-year posttreatment. The overall diversity of this division decreased upon antibiotic treatment and remained reduced during the entire 2 years of the study. The authors also show that the dominant community members changed markedly in relative abundance during the first 3 weeks of the posttreatment, suggesting that these effects were not exclusive to the rest of the microbiota.

We draw attention to these dynamics here to simply point out that the gut microbiota changes markedly during and after normal therapeutic courses of antibiotics and that host susceptibility to subsequent infection is increased as a result. We now turn to specific clinical presentations that result from antibiotic treatment of human patients and follow with a discussion on a microbial ecology approach to these diseases.

## 5. ANTIBIOTIC-ASSOCIATED DIARRHEA AND *C. DIFFICILE*

Patients undergoing antibiotic treatment often develop diarrhea (antibiotic-associated diarrhea or AAD) as a side effect of therapy. Approximately, 5%–25% of patients on antibiotic therapy develop AAD, which can range from a mild, self-limiting illness to a serious and progressive pseudomembranous colitis [41, 42]. The risk of developing disease is highly variable and depends on host factors (age, diet, immune system function, etc.), the type and dose of antibiotic, and the duration of treatment. In a cohort study, Beaugerie et al. found that 17.6% (46 out of 262) of adult (≥18 years old) outpatients developed diarrhea within 14 days after the start of treatment [43]. Patients that remain in the hospital are similarly affected. According to a prospective study of hospitalized patients in Sweden, 12% (294 out of 2 462) of patients ≥12 years old developed diarrhea within 45 days after the start of treatment [44]. However, certain patient populations in the hospital appear to be at an elevated risk as 60% (9 out of 15) of individuals (ages 37–79) enrolled in a cohort study of intensive care units developed diarrhea within the first week after of antibiotic treatment [45]. These data illustrate that diarrhea is a common complication of antibiotic use and suggest that critically ill patients are exquisitely susceptible to AAD.

An etiologic agent is not necessary for AAD, as certain drugs can cause gastrointestinal dysfunction directly [42]. A distinction can, then, be made between pathogen-associated and pathogen-independent AAD in that Koch's postulates are not met in the classical sense. For example, if the bacteria responsible for breaking down fermentable starches in the colon are eliminated by the effect of an antibiotic, an osmotic diarrhea may present. In this scenario, the community and not a defined pathogen is responsible for the disease etiology. To our knowledge, however, replicating the disease in an otherwise naïve individual by establishing the “pathogenic community” has not been shown.

A number of opportunistic pathogens can cause disease during antibiotic therapy, including *Salmonella* spp., *Clostridium perfringens*, *Klebsiella oxytoca*, *S. aureus*, *Candida albicans*, and *C. difficile*. Of these, *C. difficile* is the most common cause of pathogen-associated AAD (15%–25%), the most common cause of severe disease, and it causes nearly all cases of nosocomial pseudomembranous colitis [46]. *C. difficile* is an anaerobic, spore forming bacterium that is commonly found in soil, humans, and animals [47]. This toxigenic gram-positive bacillus is asymptomatically carried by 1%–3% of the human population, but is more prevalent among infants [23], hospitalized patients (55.4% of the hospital population in the Swedish AAD cohort study mentioned above [44] were positive for *C. difficile* toxin), older (≥60 years) patients [47–49], and healthcare personnel that care for patients being treated with antibiotics [50]. This pathogen can cause disease in nonhospitalized patients [51], where the main risk factors are antibiotic therapy, proton pump inhibitors, and the use of histamine-2-receptor antagonists [52].

Pseudomembranous colitis in the distal colon and rectum is fatal in 6%–30% of cases [47]. Disease onset occurs several days to several weeks after initial antibiotic treatment and certain drugs, such as clindamycin, cephalosporins, fluoroquinolones, and *β*-lactams, are associated with greater risk of CDAD [46, 53]. Oral antibiotic therapies with vancomycin, metronidazole, bacitracin, teicoplanin, and fucidin have been shown to be an effective initial treatments for CDAD [54]. A significant number (20%–35%) of patients develop recurrent illness caused by the same or different *C. difficile* strains and symptoms arise several days (usually >4) to several weeks after the apparent success of the initial antibiotic therapy [55–57].

CDAD has been a recognized health problem in the United States and many industrialized countries for more than 30 years [58], but the epidemiology of the disease is changing. The prevalence and severity (case fatality rate) of CDAD continue to increase in spite of numerous discoveries concerning its epidemiology, pathogenicity, and treatment [53, 59]. This increasing trend is associated with the emergence and spread of an epidemic strain referred to as NAP1/BI (North American pulsed-field type 1, ribotype 027, restriction endonuclease analysis type BI, toxinotype III) [47, 60]. As a result, the average inhospital cost of CDAD patients is estimated to be 54% more than non-CDAD patients in the United States, adding an overall $1.1 billion to national health care costs [61]. Length of hospital stay also increases with CDAD patients and ranges from an average of 3.6 days for the total inpatient population to 16 days for surgical inpatients [62].

## 6. THE MICROBIAL ECOLOGY APPROACH TO AAD AND CDAD

There are few data that assess changes in the human gut microbiota during the course of AAD. The only sequence-based, microbial ecology study to date followed a 39-year-old male throughout an amoxicillin-clavulanic acid treatment (875 and 125 mg, resp., 2 times daily for 10 days) for acute sinusitis [63]. The patient developed non-CDAD within 24 hours of the first dose and symptoms persisted until 4 days after the final dose. Stool samples were taken 12 hours after the first dose (day 0), 4 days into the 10-day regime (day 4), and at 2 weeks following the final dose (day 24). A total of 84, 74, and 84 randomly cloned 16S rRNA genes were sequenced from each sample, respectively.

At 4 days into the amoxicillin-clavulanic acid therapy, the gut microbiota of this individual was markedly shifted. Representation of the Bacteroides group went from exclusively *B. fragilis* on day 0 to almost all *B. distasonis* on day 4. There was also a dramatic outgrowth of Enterobacteriaciae (most likely *E. coli*). Lastly, all members of the Clostridial rRNA cluster XIVa and Bifidobacteria groups (32% of the all sequences on day 0) were lost or below the detection limit.

Two weeks after the last dose of antibiotic, the microbiota appeared to be recovering to day 0 composition. The *B. fragilis* and Clostridial rRNA cluster XIVa groups rebounded, while *B. distasonis* and Enterobacteriacea groups were drastically decreased or undetected. Interestingly, members of the Clostridial rRNA cluster IV group were relatively unaffected by the antibiotic treatment and were sampled at roughly even numbers on all 3 sampling days. In contrast, members of the Bifidobacteria group were lost or below detection by day 4 and remained so at day 24. These data suggest that (i) the composition of the gut bacterial community is dramatically shifted during antibiotic therapy, (ii) that resiliency to this drug's effects is group specific, and (iii) that it may require an extended period of time for the microbiota to recover to the prestressed composition, if at all. More data are needed to adequately assess the rate and extent of recovery from this and other antibiotics and to assess how variable these effects are in the human population.

The association between CDAD and perturbations of the gut microbiota is well established but poorly understood. For example, animal (hamster and mouse) and in vitro models show antagonism between conventional microbiota and *C. difficile* population growth [64]. These findings help to explain the success of bacteriotherapy for recurrent-CDAD, where the disease was resolved by rectal instillation of donor stool [65, 66]. However, the use of probiotics and synthetic mixtures of bacteria has had limited success [67] and is not currently efficacious as alternative therapies. The hope is that a better understanding of the complexity of this system during CDAD infection will lead to defined manipulations of patient microbiota that will both prevent establishment of this pathogen and treat acute disease.

To this end, Chang et al. recently applied the same approach discussed above (16S rRNA gene sequencing) to 7 patients with initial (*n* = 3) and recurrent (*n* = 4) CDAD and 3 control individuals from an outpatient clinic [68]. Species level identity based on 97% nucleotide similarity was determined for 125–184 16S rRNA genes per individual. To gain insight into the overall bacterial diversity of each patient's fecal microbiota, rarefaction curves were generated from these sequence data. Rarefaction is a method of generating idealized taxonomic “collectors curves” from community data through data resampling [69]. The shape of the rarefaction curves is then indicative of the overall complexity of the microbiota in each community, allowing comparison of the diversity of each patient's fecal microbiota.

At this level of community sampling, inferences were restricted to the most abundant members. However, and without exception, the microbiota from control and initial CDAD patients was more complex than the microbiota from recurrent CDAD patients. Furthermore, the authors were able to combine these data with those from the non-CDAD-AAD patient [63] to show a clear association between microbiota complexity and disease outcome (i.e., Controls > AAD > initial CDAD ≫ recurrent CDAD). This study not only provides a support for the barrier function against *C. difficile* establishment and disease, but also because the sequences represent actual organisms, these data can be used to identify potentially useful antagonistic relationships in the community.

The 16S rRNA clone library approach is useful to study interesting symbiotic associations in bacterial communities. This and other techniques may also be useful in predicting clinical outcomes based on their association with specific consortia of bacteria. To do so requires a novel conceptualization of the disease process in that one particular organism is not necessarily defined as the causative agent, but rather the entire community is involved in causing the outcome. There is little information available to generate these types of risk models, but the clinical potential in using microbial ecological inferences to guide therapies (i.e., tapering antibiotic treatments, probiotics, etc.) and prevention certainly warrants further investigation.

## 7. CONCLUSIONS AND FUTURE DIRECTION

Comparative studies that use microbial ecology techniques to analyze temporally sampled patients and control individuals are a promising approach to complex disease research. Traditional culture-based methods continue to be the gold standard for disease diagnostics, but this approach can only detect organisms that are easy to isolate and have simple metabolic requirements. Since the vast majority of the human gut microbiota is currently noncultivable, a nonculture-based approach may be more useful for the diagnosis and prediction of clinical outcomes [70]. Analyzing the metagenome is such an approach and can be used to identify members of complex bacterial communities based on nucleotide variability in conserved genes [70, 71]. New technologies, such as pyrosequencing, have recently become available and attain the high throughput and resolution required to make detailed community comparisons based on more than one locus. An added benefit of these technologies is that reagents and chemistries are constantly being re-engineered so that efficiency is maximized at lower cost.
